# Wall shear stress analysis using 17.6 Tesla MRI: A longitudinal study in ApoE^-/-^ mice with histological analysis

**DOI:** 10.1371/journal.pone.0238112

**Published:** 2020-08-28

**Authors:** Katharina A. Riedl, Thomas Kampf, Volker Herold, Volker C. Behr, Wolfgang R. Bauer

**Affiliations:** 1 Department of Experimental Physics V, University of Würzburg, Würzburg, Germany; 2 Department of Cardiology, University Heart & Vascular Center Hamburg, Hamburg, Germany; 3 Department of Diagnostic and Interventional Neuroradiology, University Hospital Würzburg, Würzburg, Germany; 4 Department of Internal Medicine I, University Hospital Würzburg, Würzburg, Germany; Max Delbruck Centrum fur Molekulare Medizin Berlin Buch, GERMANY

## Abstract

This longitudinal study was performed to evaluate the feasibility of detecting the interaction between wall shear stress (WSS) and plaque development. 20 ApoE^-/-^ mice were separated in 12 mice with Western Diet and 8 mice with Chow Diet. Magnetic resonance (MR) scans at 17.6 Tesla and histological analysis were performed after one week, eight and twelve weeks. All *in vivo* MR measurements were acquired using a flow sensitive phase contrast method for determining vectorial flow. Histological sections were stained with Hematoxylin and Eosin, Elastica van Gieson and CD68 staining. Data analysis was performed using Ensight and a Matlab-based “Flow Tool”. The body weight of ApoE^-/-^ mice increased significantly over 12 weeks. WSS values increased in the Western Diet group over the time period; in contrast, in the Chow Diet group the values decreased from the first to the second measurement point. Western Diet mice showed small plaque formations with elastin fragmentations after 8 weeks and big plaque formations after 12 weeks; Chow Diet mice showed a few elastin fragmentations after 8 weeks and small plaque formations after 12 weeks. Favored by high-fat diet, plaque formation results in higher values of WSS. With wall shear stress being a known predictor for atherosclerotic plaque development, ultra highfield MRI can serve as a tool for studying the causes and beginnings of atherosclerosis.

## Introduction

Atherosclerosis is a pathological chronic inflammatory process in the arterial wall especially of the endothelial cells affecting cardiovascular disease [1]. Human atherosclerotic plaque formation in the coronary arteries is associated a 2.5fold higher risk to die due to cardiac events [2]. Hemodynamic parameters like wall shear stress (WSS) or pulse wave velocity (PWV) can influence the regional compliance of the vessel and is discussed as an essential predictor of the atherosclerotic plaque formation [3–7]. A change of the shear stress at the aortal wall is discussed as a predictor for the development of atherosclerotic plaque formation especially at the branch of the aorta and the inner curvature of the aortic arch [8–11]. The WSS is a force per area exerted on a surface by a liquid flowing parallel to this surface measured in Nm2 [12, 13].

The WSS is defined as

$$\vec{\tau }=2\eta \overset{⃡}{\varepsilon }∙\vec{n}$$ (1)

with the deformation tensor

$$\varepsilon_{ij}=\frac{1}{2}∙\left(\frac{\partial v_{i}}{\partial x_{j}}+\frac{\partial v_{j}}{\partial x_{i}}\right),i,j=1,2,3$$ (2)

with $\vec{\tau }$ = WSS, *η* = viscosity, $\vec{n}$ = inward unit normal of the surface, *x*_*i*,*j*_ = spatial dimensions and *v*_*i*,*j*_ = velocity components [12, 14].

Previous studies described the possibility of evaluating the WSS via 1.5 and 3 Tesla MRI in human vessels with contrast agent and without a longitudinal MR follow up or histological analysis [12, 15]. Furthermore, some studies performed WSS measurements also in murine vessels [16, 17], and for example Zhao et al. measured the WSS via a phase contrast method in the murine abdominal aorta in even 9 month old mice [18]. For evaluating the atherosclerotic plaque development and the WSS over a longer period in time, Apolipoprotein E-deficient (ApoE^-/-^) mice are a feasible model due to their spontaneous atherosclerotic plaque development [19, 20]. The longitudinal *in vivo* murine WSS evaluation using 17.6 Tesla magnetic resonance imaging (MRI) has previously not been explored.

Thus, the purpose of this study was performing a longitudinal analysis to evaluate the feasibility of detecting the interaction between the wall shear stress and the beginning atherosclerotic plaque development in the murine aortic arch using 17.6 Tesla MRI with histological analysis.

## Materials and methods

### Animal protocol

**Fig 1:**
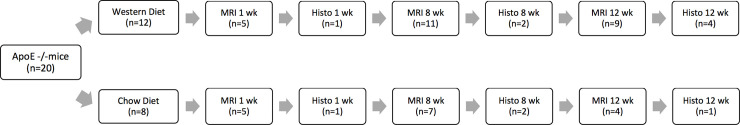
Flow chart of the protocol of the longitudinal mice study. MRI = magnet resonance imaging, Histo = histological analysis, wk = weeks.

This longitudinal animal study was performed with 20 eight-week-old female ApoE^-/-^ mice (Charles River Laboratories, Sulzfeld, Germany) at the Physical Institute Würzburg (Germany). These mice were randomly separated in two groups consisting of twelve ApoE^-/-^ mice placed on atherogenic Western Diet (E15721-347, ssniff special diets GmbH, Soest, Germany) with a fat content of 21.2% and eight ApoE^-/-^ mice placed on Chow Diet (V1534-000, ssniff special diets GmbH, Soest, Germany) with a fat content of 3.3%, starting at the age of eight weeks. The unequal group sizes were chosen to compensate for the higher expected mortality in the group placed on Western Diet. Western Diet accelerates the formation of atherosclerosis in ApoE^-/-^ mice [20]. Magnetic resonance (MR) scans and histological analysis were performed after one to two week, eight weeks, and twelve weeks according to the study plan (Fig 1, S1 Table). This study was designed as a feasibility study for sequential MR WSS measurements in established atherosclerosis. Therefore, only a limited number of mice were allowed for baseline measurements in accordance to the animal protection (S1 Table).

### Animal handling

The mice were maintained on a 12h-light- and 12h-dark-cycle at 20–22°C indoor temperature and a relative humidity of (55 ± 10)% and were provided with water access and food (one group with Western Diet and one group with Chow Diet) *ad libitum*. Mice were group housed (2–4 mice per cage) and prior to experiments step-by-step acclimatized to the laboratory environment.

All examinations were performed under isoflurane narcosis to prevent suffering. Isoflurane was used for anesthesia due to the smallest effect on the hemodynamic parameters in contrast to other anesthetics as demonstrated by Janssen et al. [21]. The mice were induced into anesthesia at dose of 3–4 Vol.% isoflurane, then maintained by continuous inhalation of 1.5–2 Vol.% isoflurane and 2 Lmin O_2_ during spontaneous breathing. Mouse physiology was continuously monitored using a breathing and electrocardiographic (ECG) monitoring unit during the measurement. By individually adjusting anesthesia for each mouse to maintain ECG periods of about 110 ms and respiratory periods of about 1200 ms a comparable depth of anesthesia in all experiments was ensured. Body temperature was maintained by using a heating bed during animal preparation and by adjusting the temperature of the gradient cooling unit to 36°C during the MR measurements.

The mice had been painlessly euthanized under overdose isoflurane anesthesia with a following exsanguination according to Annex IV ‘Methods of killing animals’ of the Directives of the European Parliament and of the Council on the protection of animals used for scientific purposes [22].

All experimental procedures were in accordance with the institutional and internationally recognized guidelines [22] and were approved by the Regierung von Unterfranken (Government of Lower Franconia), Würzburg, Germany, to comply with German animal protection law under reference number 55.2.-2531.01-23/11.

### Magnetic Resonance Imaging (MRI)

**Fig 2:**
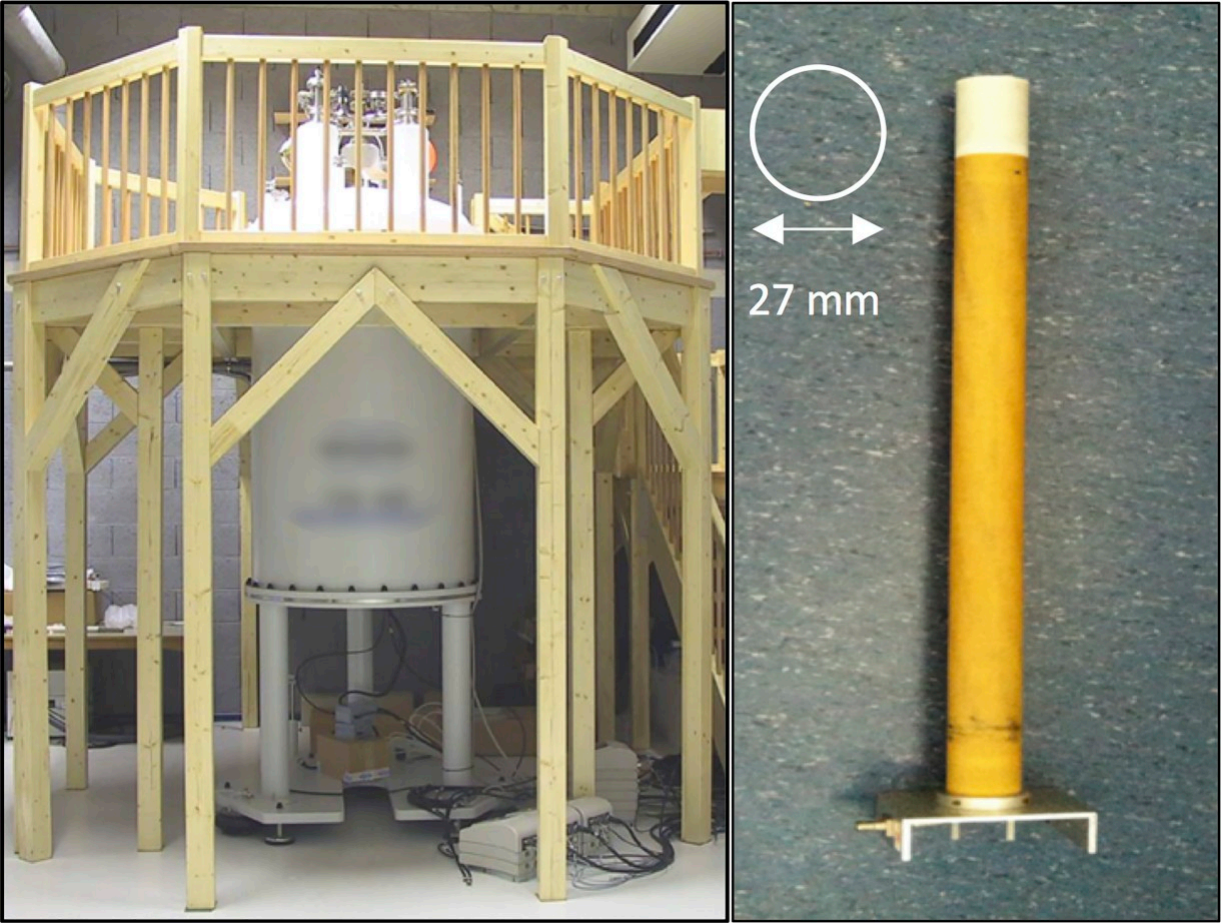
MRI hardware. Ultra highfield Bruker 17.6 Tesla MRI at Würzburg (left panel). Birdcage resonator with an inner diameter of 27 mm (right panel).

A 2D gradient echo imaging method with a 3D phase contrast flow encoding [23] was validated and optimized using an ultrahigh field 17.6 Tesla MRI (Avance 750WB) with an 89 mm vertical bore operated by ParaVision 4.0 (Bruker BioSpin, Rheinstetten, Germany). The spectrometer is equipped with a 1000 mTm gradient unit offering a 40 mm bore for rf resonators and samples. A 27 mm inner diameter custom-built birdcage resonator was employed in all imaging experiments (Fig 2). This setup enables the analysis of blood flow velocity in very narrow vessels.

Prior to the longitudinal *in vivo* study measurement procedures were established using phantoms and WT C57Bl/6 mice. As phantoms served tubes with a diameter of 4 mm and of 1 mm, respectively, with an aqueous solution of copper sulfate (1.25 gml) circulating at 1 cms driven by a gear pump. Volumetric flow rate was determined by measuring the water volume drained from the tube per time and compared with the results obtained from MRI measurements.

**Fig 3:**
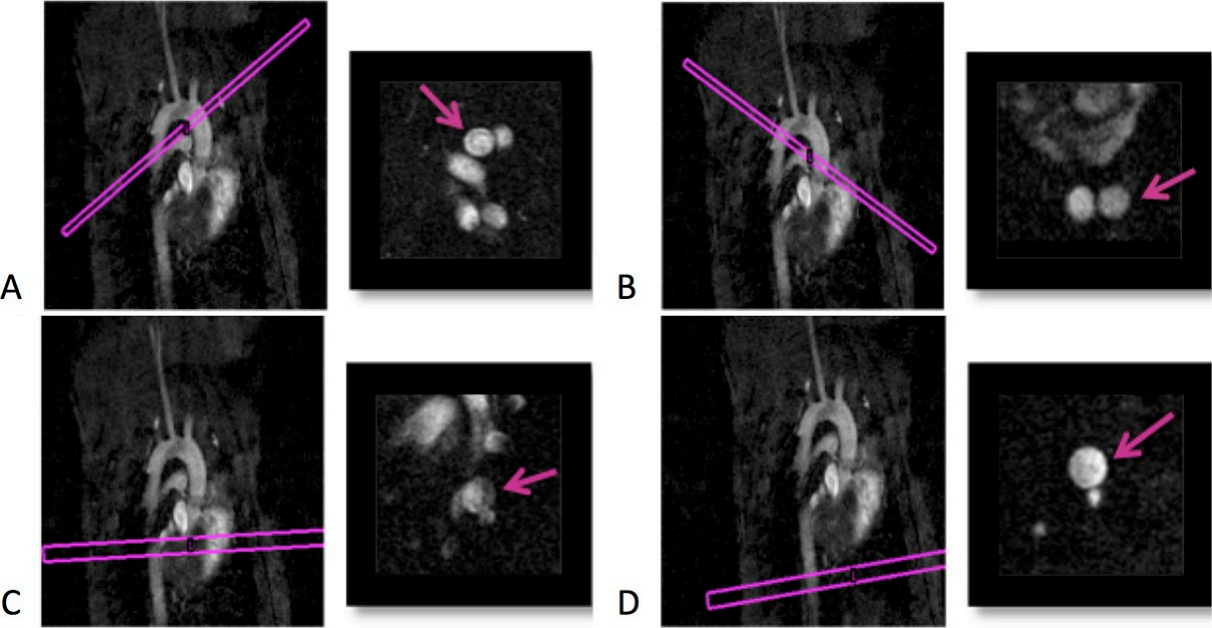
Morphological MR planes. A. before the Truncus brachiocephalicus (MR plane 1), B. after the A. subclavia sinistra (MR plane 2), C. and D. in the thoracic part of the aorta (MR plane 3 and 4); Aorta indicated by purple arrows.

For *in vivo* measurements mice were placed head first in the resonator and then the entire setup was inserted into the scanner from the bottom. Measurements were performed breath triggered with a pneumatic sensing balloon at the mouse chest and a trigger unit (Rapid Biomedical, Rimpar, Germany) (S1 Fig). All *in vivo* MR measurements were acquired with a field-of-view of 25 mm x 20 mm at a matrix size of 250 x 200 and a slice thickness of 0.5 mm using a 35° flip angle, an echo time (TE) of 1.8 ms, a repetition time (TR) of 5 ms, 4 averages and 35 frames. The maximum encodable velocity was 167 cms, the temporal resolution 5 ms and the total acquisition time about 10 minutes. The four MR planes were located in the thoracic part of the aorta before the Truncus brachiocephalicus, after the Arteria subclavia sinistra and in the thoracic aorta, orthogonal to blood flow (Fig 3).

The optimal position of the mouse in the resonator and the MR plane orthogonal to the blood flow and the wall of the aorta was important to achieve comparable and reproducible MR scans for the following analysis of the flow parameters. All MR scans were performed without using contrast agents or filters in postprocessing.

### WSS analysis

Data was imported into Matlab (The MathWorks, Natick, MA, USA) and exported to Ensight (CEI, Apex, NC, United States), where manual segmentation of the relevant anatomic region was performed [24]. In a following step these data were processed using the Matlab-based “Flow Tool” (Department of Radiology, Medical Physics, University of Freiburg, Freiburg, Germany). Information on the internal algorithms of “Flow Tool” are described in [12]. In this tool the individual vessel cross sections were manually segmented using a B-Spline interpolation on the morphological data. Subsequently the tool computed from the corresponding flow data velocity maps for the selected MR planes and based on these the WSS. For blood viscosity and density the preset values of “Flow Tool” (viscosity: 4.5∙10^−3^ Nsm2 and density: 1055 kgm3) were used [12, 25]. All WSS data in this study are presented as mean WSS values averaged over the whole circumference.

### Histological analysis

Histological analysis was conducted at the first, second and third MR measurement point. The aortae were excised and perfused with Tissue Tec O.C.T. Compound (Sakura Finetek Europe B.V., Alphen aan den Rijn, The Netherlands) and stored at -80°C. Serial, transversally cut 8 *μ*m sections of the thoracic aorta were collected at -21°C with a cryotom (Leica CM 1850, Leica Biosystems, Nussloch, Germany). The histological section planes were stained with Hematoxylin and Eosin (HE), Elastica van Gieson and CD68 staining (Merck KGaA, Darmstadt, Germany). For visualization of the elastin laminae and fragmentations the sections were stained with an Elastica van Gieson staining kit. The CD68 coloring (MCA 1957. AbD Serotec, Oxford, UK) is an immunohistochemical staining for macrophages and was performed in an ApoE^-/-^ mouse after twelve weeks Western Diet.

### Statistical analysis and WSS analysis

The continuous variables were described using mean ± standard deviation (SD) (range) or median (interquartile range, range). Shapiro-Wilk test was used for testing the normal distribution of the data and Levene’s test for homogeneity of variances. Differences between Western Diet group and Chow Diet group were tested using the Student’s t-test with unequal variables for normally distributed data or using the Mann-Whitney U test for not normally distributed data. Changes over time in the individual groups were not evaluated due to the low numbers of mice in each group. A p value ≤0.05 was considered statistically significant. All statistics were done with SPSS version 25 (^©^IBM corporation and its licensors 1989, 2017).

## Results

### Phantom measurements

Using the flow phantom with a diameter of 4 mm, the volume per time was (2.911 ± 0.054) mls and the flow via MRI was (2.979 ± 0.101) mls (S2 Table); in the flow phantom with a diameter of 1 mm, the volume per time was (0.220 ± 0.003) mls and the flow via MRI (0.215 ± 0.003) mls (S3 Table). This comparison showed a very good agreement of both methods.

### Study population

The body weight of all 20 ApoE^-/-^ mice with Western Diet and Chow Diet increased significantly over the study period of 12 weeks (Table 1, S2 Fig).

**Table 1:** Weight of the ApoE^-/-^ mice according to the diet type.

		Western Diet (n = 12)	Chow Diet (n = 8)	p Value^a^
Body weight in g	1 week	20.3 ± 0.3 (20.0–20.8) (n = 5)	17.4 ± 1.1 (15.7–18.4) (n = 5)	0.003
	8 weeks	23.6 ± 1.2 (21.0–25.8) (n = 11)	21.3 ± 1.1 (19.9–22.5) (n = 7)	0.001
	12 weeks	26.1 ± 2.0 (22.8–28.8) (n = 9)	22.5 ± 1.3 (21.2–24.2) (n = 4)	0.004

Values are mean ± SD (range).

^a^Comparison between the groups of Western Diet and Chow Diet.

### Wall shear stress (WSS)

**Fig 4:**
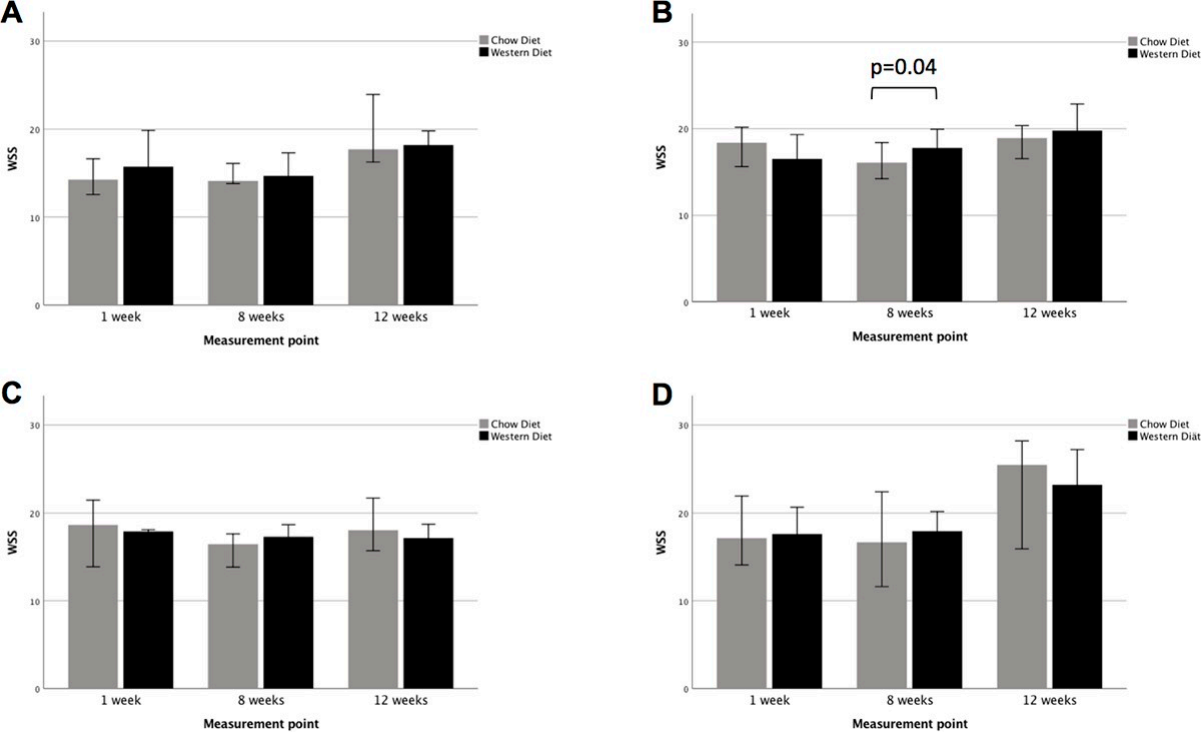
WSS (N/m^2^) at three measurement points in mice with Western Diet (left panel) and Chow Diet (right panel). A: MR plane 1, B: MR plane 2, C: MR plane 3, D: MR plane 4. Values are median with 95% confidence interval. WSS with the unit N/m^2^.

WSS was evaluated at four MR planes illustrated in Fig 3. Conclusive WSS results were available in 157 (96%) MR scans. Possible reasons for inconclusive results were impaired *in vivo* measuring conditions, e.g. change of the breathing or heart rate or change of the position of the mouse in the resonator. Exemplary MR flow profiles of a cross section are demonstrated in S3 Fig. Comparing the WSS values at the first and the last measurement point the values increased in all MR planes in both groups, Western Diet and Chow Diet, excluding MR plane 3. The WSS values increased tendentially in the Western Diet group over the study period. In contrast the WSS values of the Chow Diet mice decreased tendentially comparing the first and the second measurement point. There was a tendency that WSS in the Western Diet group at MR plane 1 from the second to the third measurement point increased (14.7 Nm2 vs. 18.2 Nm2) and in the Chow Diet group at MR plane 2 and 4 between the first and the second measurement point (18.4 Nm2 vs. 16.1 Nm2 and 17.1 Nm2 vs. 16.7 Nm2) decreased; however, it was not statistically significant. A significant difference of the WSS values was observed at the second measurement point at MR plane 2 between the Western Diet and the Chow Diet group (17.8 Nm2 vs. 16.1 Nm2, p = 0.04) (Table 2, Fig 4). The development of WSS in all individual mice over the 12 weeks can be found in S4 Fig.

**Table 2:** WSS values according to the diet type.

	Measurement point	WSS (N/m^2^)		p Value^a^
		Western Diet (n = 12)	Chow Diet (n = 8)	
MR plane 1	1 week	15.7 (13.6–18.3, 13.2–19.9) (n = 5)	14.3 (13.4–15.7, 12.6–16.6) (n = 5)	0.29
	8 weeks	14.7 (14.3–16.9, 13.7–20.9) (n = 11)	14.2 (13.9–15.3, 13.8–16.1) (n = 6)	0.27
	12 weeks	18.2 (17.8–18.6, 16.6–19.8) (n = 8)	17.7 (15.9–22.7, 15.8–23.9) (n = 4)	0.87
MR plane 2	1 week	16.5 (15.1–19.0, 15.0–19.3) (n = 5)	18.4 (16.8–19.5, 15.6–20.2) (n = 5)	0.32
	8 weeks	17.8 (16.6–19.5, 14.0–20.3) (n = 10)	16.1 (14.4–16.9, 14.2–18.4) (n = 7)	0.04
	12 weeks	19.8 (17.3–22.9, 16.8–23.8) (n = 9)	18.9 (16.8–20.4, 16.5–20.4) (n = 4)	0.30
MR plane 3	1 week	17.9 (14.7–18.1, 14.3–18.1) (n = 5)	18.7 (14.5–21.4, 13.9–21.5) (n = 4)	0.50
	8 weeks	17.3 (14.2–18.6, 12.7–23.6) (n = 10)	16.4 (13.8–16.8, 13.8–17.6) (n = 7)	0.28
	12 weeks	17.1 (15.7–18.6, 14.0–20.0) (n = 9)	18.0 (n = 4)	0.53
MR plane 4	1 week	17.6 (14.5–19.3, 14.3–20.7) (n = 5)	17.1 (15.3–20.8, 14.1–21.9) (n = 5)	0.66
	8 weeks	17.9 (15.9–18.8, 11.6–22.0) (n = 11)	16.7 (15.1–18.8, 11.6–22.4) (n = 7)	0.71
	12 weeks	23.2 (17.6–26.6, 14.4–29.0) (n = 9)	25.5 (n = 3)	0.81

Values are median (interquartile range, range). WSS = wall shear stress, MR = magnetic resonance. WSS with the unit N/m^2^.

^a^ Comparison between the groups of Western Diet and Chow Diet.

### Histological analysis

**Fig 5:**
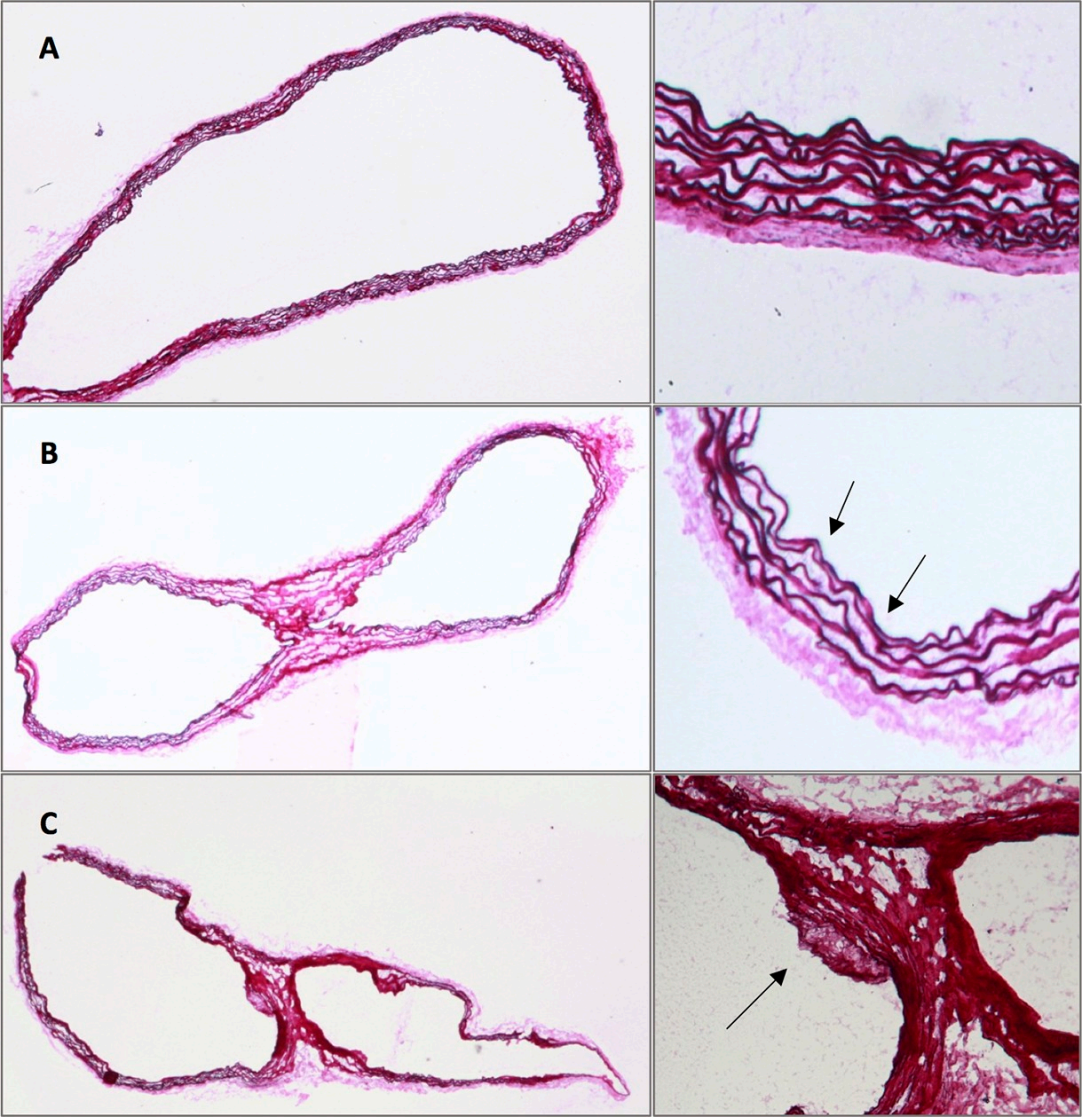
Elastica van Gieson analysis in the Western Diet group. Comparison at the first (A), second (B) and third (C) measurement point; Elastin fragmentations and plaques indicated by black arrows.

**Fig 6:**
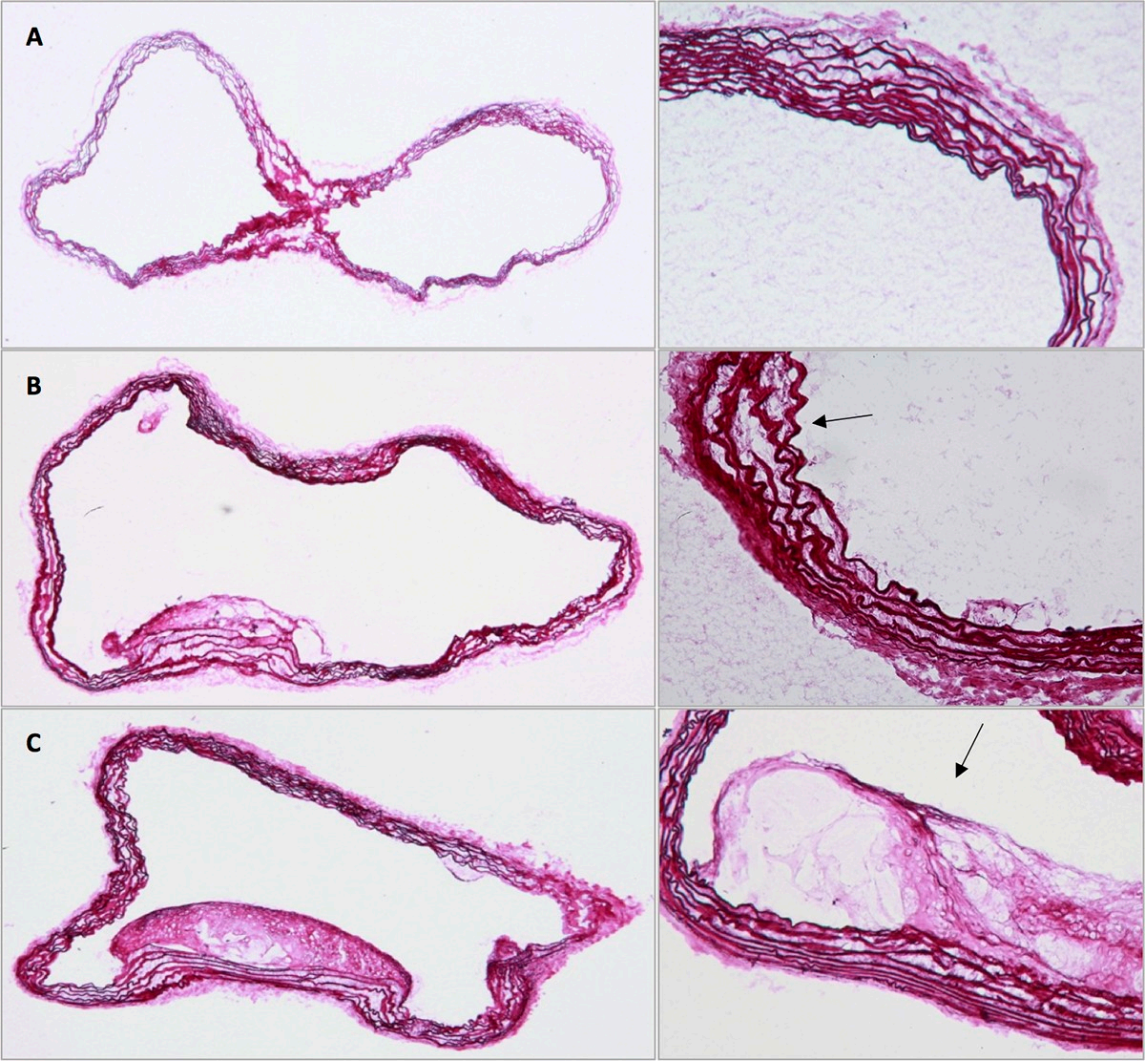
Elastica van Gieson analysis in the Chow Diet group. Comparison at the first (A), second (B) and third (C) measurement point; Elastin fragmentations and plaques indicated by black arrows.

**Fig 7:**
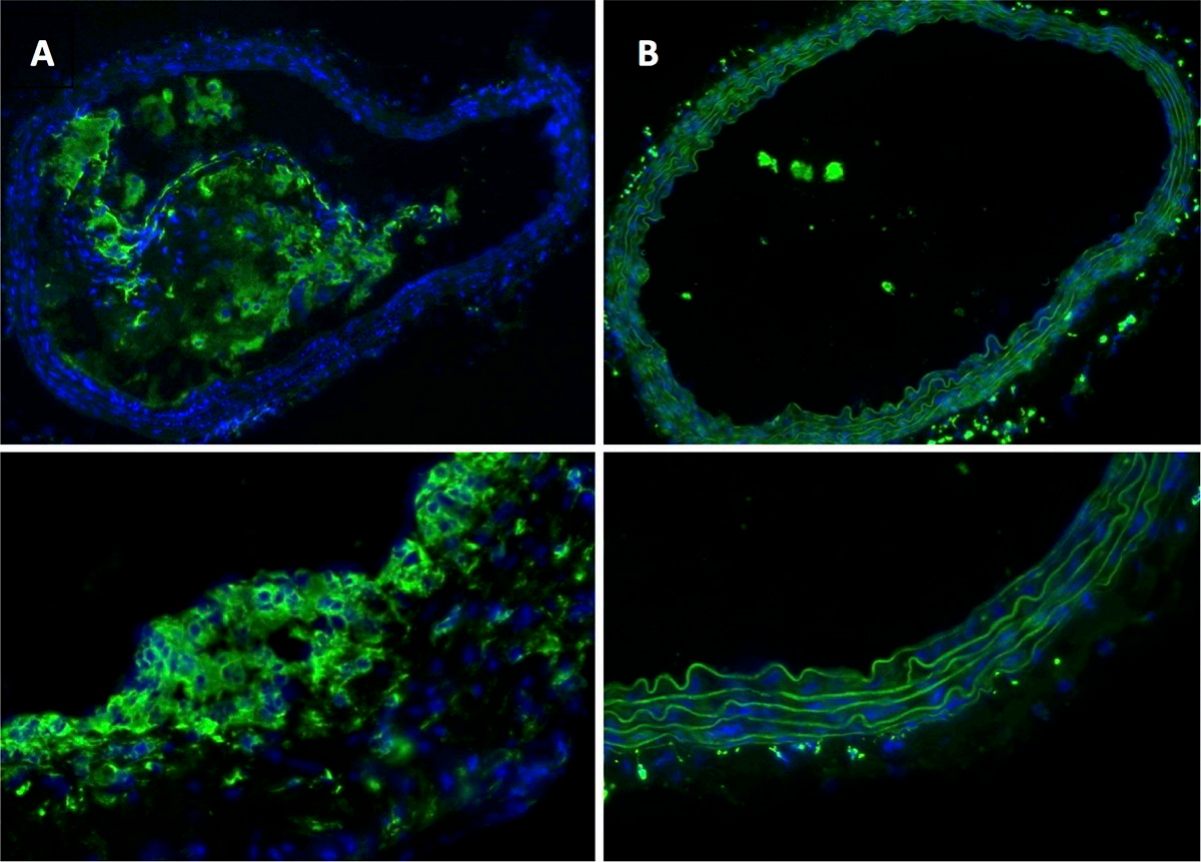
CD68 staining in ApoE^-/-^ mice after 12 weeks with Western Diet. (A) without plaque formation in the aortic arch, (B) with big plaque formation with macrophages inside in the thoracic part of the aorta.

The histological analysis was performed in mice in the Western Diet group and the Chow Diet group at each measurement point after one week, eight weeks, and twelve weeks. Spatial correlation between histological analysis and MRI was established by observation and comparison of morphological landmarks in both modalities. In the group of mice with Western Diet the histological analysis showed a small plaque formation with elastin fragmentations after eight weeks and big plaque formations and severe elastin fragmentations after twelve weeks. In contrast, in the group of Chow Diet mice after eight weeks there were only a few elastin fragmentations but no plaque formation. Only at the third measurement point the histological analysis could exhibit bigger plaque formations (Figs 5 and 6, Table 3). The plaque formations occurred especially at the inner curvature of the aortic arch and at the bifurcations in all mice (S5 Fig). The CD68 staining of ApoE^-/-^ mice of the Western Diet group after 12 weeks demonstrated the rate of macrophages inside the plaque formation in the aortic arch (Fig 7).

**Table 3:** Histological findings according to the diet type and the examination point of time.

Diet type	Lesion type	1 week	8 weeks	12 weeks
Western Diet	No lesion	+++		
	Elastin fragmentation	+	++	+++
	Small plaque formation		++	+++
	Big plaque formation		+	++
Chow Diet	No lesion	+++	++	
	Elastin fragmentation		+	++
	Small plaque formation			+
	Big plaque formation			

+ = low manifestation, ++ = intermediate manifestation, +++ = high manifestation.

## Discussion

In this longitudinal study MR based WSS measurements were feasible in ApoE^-/-^ mice via 17.6 Tesla ultra highfield MRI using a 2D gradient echo imaging method with a 3D phase contrast flow encoding. ApoE^-/-^ mice tend to develop plaque formations in different manifestation depending on the respective diet types [26]. This study extends previous findings by demonstrating increasing plaque formations over a time period of 12 weeks with a higher severity in the group fed with Western Diet. The histological analysis found the majority of the plaques at the inner curvature of the aortic arch, which has also previously been described [6, 10, 17]. ApoE^-/-^ mice were used for both groups to achieve a good comparability of the aortic anatomy [27]. The strength of this study is the direct comparability of the MR flow parameters and histological analysis at each measurement point in contrast to human WSS evaluations [15, 28]. The WSS values increased with plaque formation in the Western Diet group [28]. In the group of mice fed with Chow Diet the WSS decreased at the second measurement point and increased at the third measurement point. The change from the high cholesterol breed feed 5K52 to the low cholesterol Chow Diet might be the reason for the decreasing WSS values [29, 30]. Wall shear stress increased over the time period of 12 weeks pooling MR plane 1 and 2 (S6 Fig). The wall area (outer diameter of the aorta minus inner diameter of the aorta) increased in the same way over this time period in the aortic arch (S6 Fig).

The change of the WSS is on the one hand the cause of the atherosclerotic plaque formation and on the other hand the consequence. A low WSS value is described as a predictor for plaque development [15, 28]. Low WSS affects an atherosclerotic plaque development [31–33], whereas the plaque itself affects a higher WSS [6]. MR plane 3, which exhibits on plaque formations, shows lower WSS values compared to the WSS values of the other MR planes. The WSS values of this study are in accordance with findings in literature [6, 34]. Previous studies showed that mice had 7 to 12fold higher WSS values compared to human WSS measurements [17, 34, 35]. Moreover, Cheng et al. could show an inverse correlation with a 7fold higher WSS comparing a mouse with a body weight of 0.03 kg with a human person with 60 kg [34]. Feintuch et al. and Trachet et al. also showed that the diameter of the aorta can also affect the WSS value with a higher WSS value in case of a smaller aortic diameter [16, 36]. These findings are in accordance with the WSS values in this study compared to previous findings from Stalder et al. [12]. The first step of longitudinal visualization of the WSS via a 2D imaging method is feasible in a high quality, thus, now the application of 3D acquisitions, as described in [37], in a longitudinal study is needed. However, the previous publication by Stalder et al. [12] showed that a 3D measuring method underestimates the WSS values in comparison to a 2D measuring method.

### Study limitation

This longitudinal animal study was performed with a small number of mice, which was reduced at each measurement point due to the histological analysis of the murine aorta. Using a 2D MR imaging method the WSS measurements could only be performed at predefined MR planes. The measurement was breath triggered depending on a constant narcosis with stable vital parameters. The histological analysis was done parallel to the tissue tec block in which the aorta was imbedded, whereby the comparison with the WSS values was possible due to the whole histological analysis of the block and the aorta.

## Conclusions

In conclusion wall shear stress measurement in the small vessels of the mouse aorta is feasible. There is a tendency towards higher values of wall shear stress in vessels affected by plaque formation. This formation is favored by a high-fat diet and can as shown by histology predominantly be detected at predilection sites at the inner curvature of the aorta. Ultra highfield MRI can therefore serve as a tool for studying the causes and beginnings of atherosclerotic plaque formation.

## Supporting information

S1 Fig *In vivo* monitoring. Pressure balloon (left panel), electrocardiogram (ECG) and breathing signal (right panel) for triggered MRI scans.
(TIFF)

S2 Fig Weight of the ApoE^-/-^ mice over the study period of twelve weeks according to the diet. (TIFF)

S3 Fig Representative flow profiles. (A) Flow profiles via FlowTool and (B) flow maps via Matlab in m/s.
(TIFF)

S4 Fig WSS values of all individual mice. W = Western Diet, C = Chow Diet, 1–4 = MR planes 1–4.
(TIFF)

S5 Fig Histological analysis (Elastica van Gieson staining) of an ApoE^-/-^ mouse at the third measurement point after 12 weeks Western Diet. (TIFF)

S6 Fig Wall area and wall shear stress in the aortic arch (MR plane 1 and 2). A: wall area (outer diameter minus inner diameter of the aorta) in mm^2^ and B: wall shear stress in N/m^2^.
(TIFF)

S1 Table Detailed overview of *in vivo* measurements of each mouse. MR = MR measurement, Histo = histological analysis with euthanization, Exitus = death during MR measurement.
(PDF)

S2 Table Velocity data of flow phantom 1 with a diameter of 4 mm. A: Volume measurement, B: MR measurement and C: representative MR scan (phantom marked with white arrows, the large object is a static cylinder filled with saline water).
(PDF)

S3 Table Velocity data of flow phantom 2 with a diameter of 1 mm. A: Volume measurement, B: MR measurement and C: representative MR scan (phantom marked with white arrow, the large object is a static cylinder filled with saline water).
(PDF)
