# Supplementary figures and images for: Wall shear stress analysis using 17.6 Tesla MRI: A longitudinal study in ApoE-/- mice with histological analysis
Source: PLoS One. 2020 Aug 28;15(8):e0238112. doi: 10.1371/journal.pone.0238112 (PMC7454980; doi:10.1371/journal.pone.0238112)

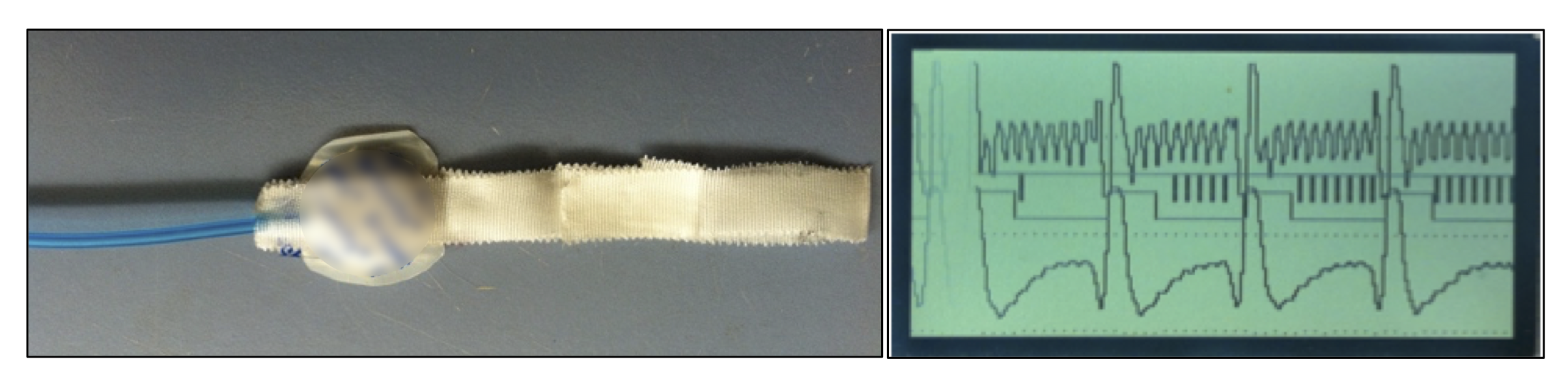

Supplement: S1 Fig — Pressure balloon (left panel), electrocardiogram (ECG) and breathing signal (right panel) for triggered MRI scans. (TIFF) [file pone.0238112.s001.tiff]

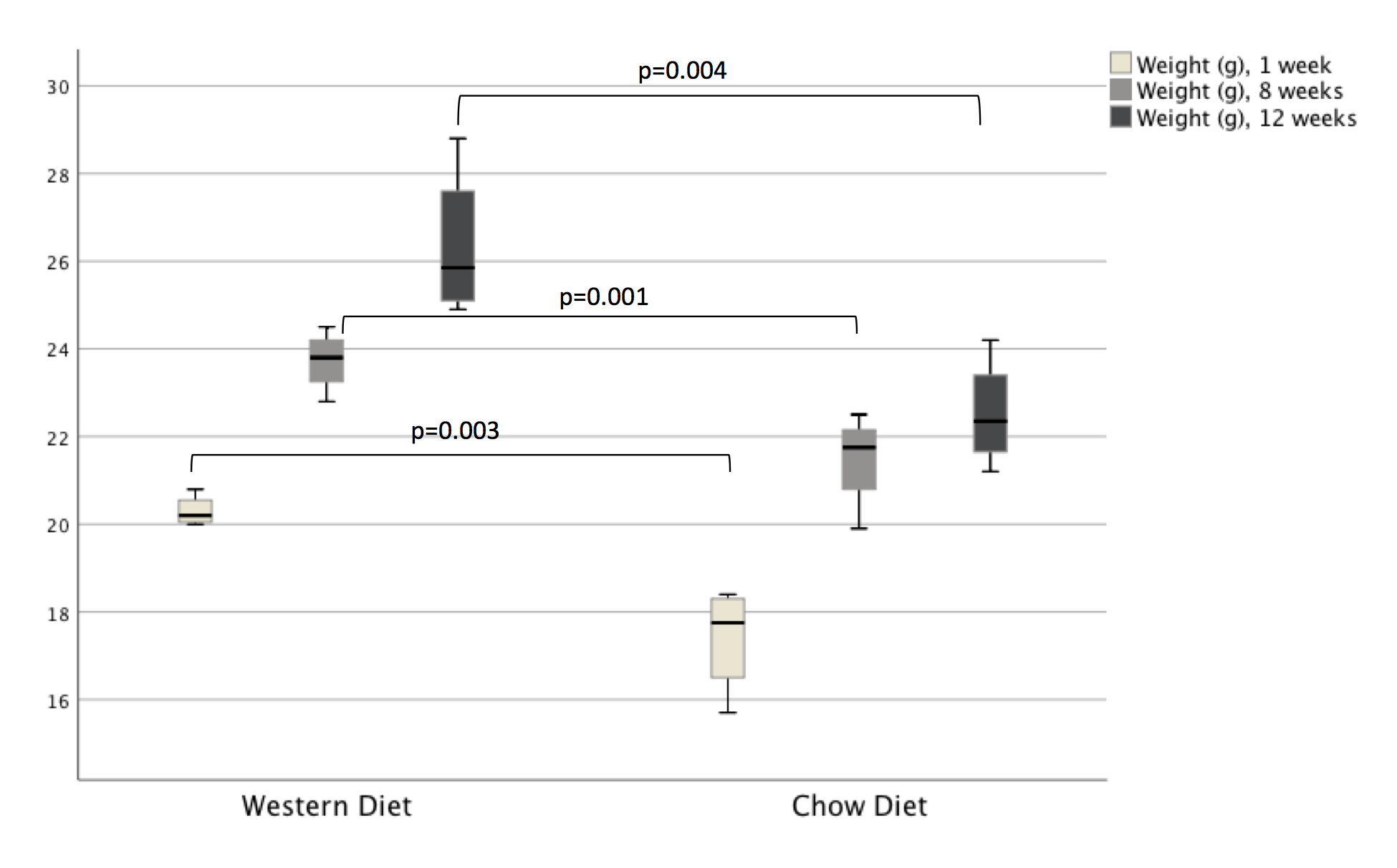

Supplement: S2 Fig — (TIFF) [file pone.0238112.s002.tiff]

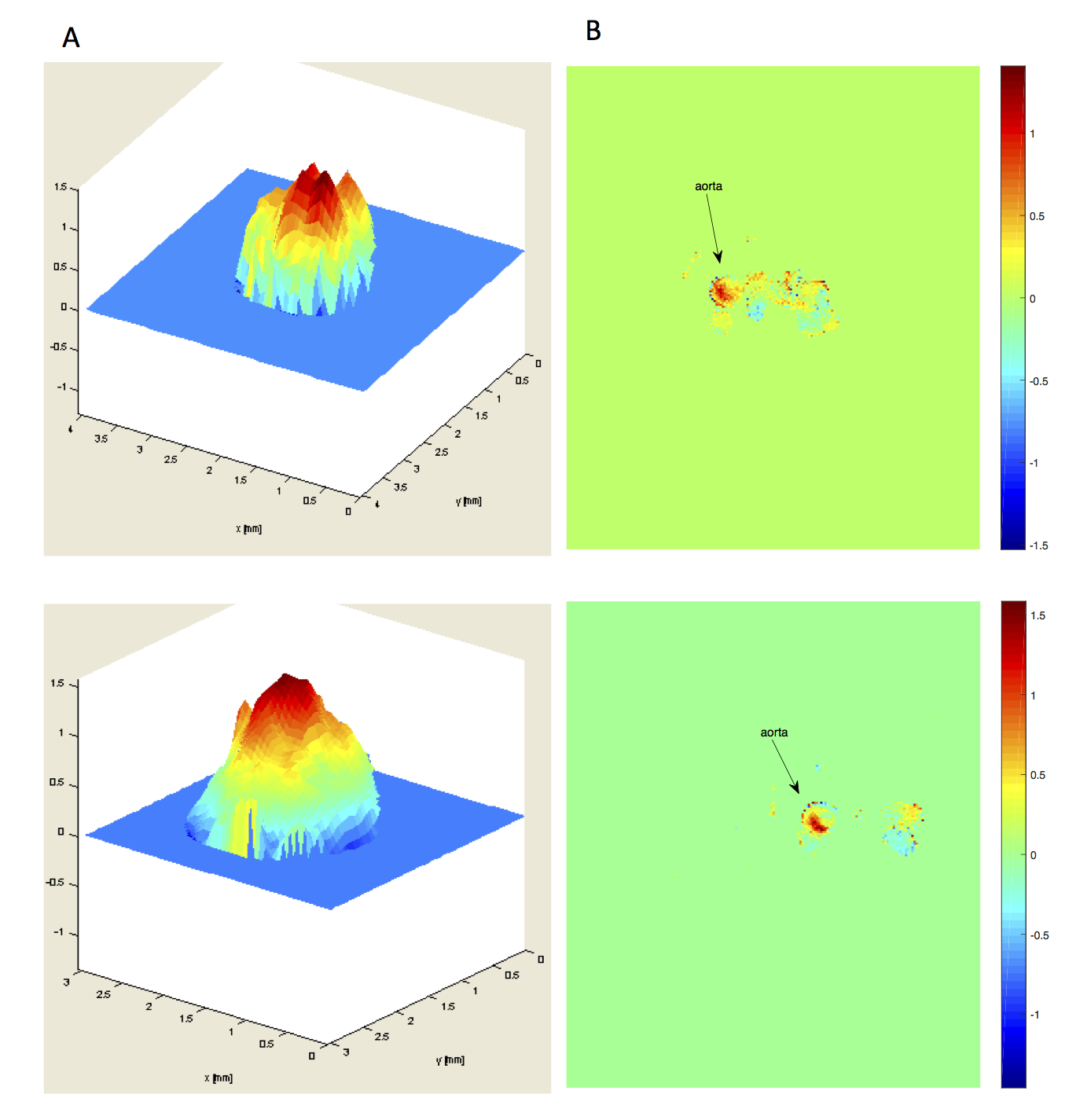

Supplement: S3 Fig — (A) Flow profiles via FlowTool and (B) flow maps via Matlab in m/s. (TIFF) [file pone.0238112.s003.tiff]

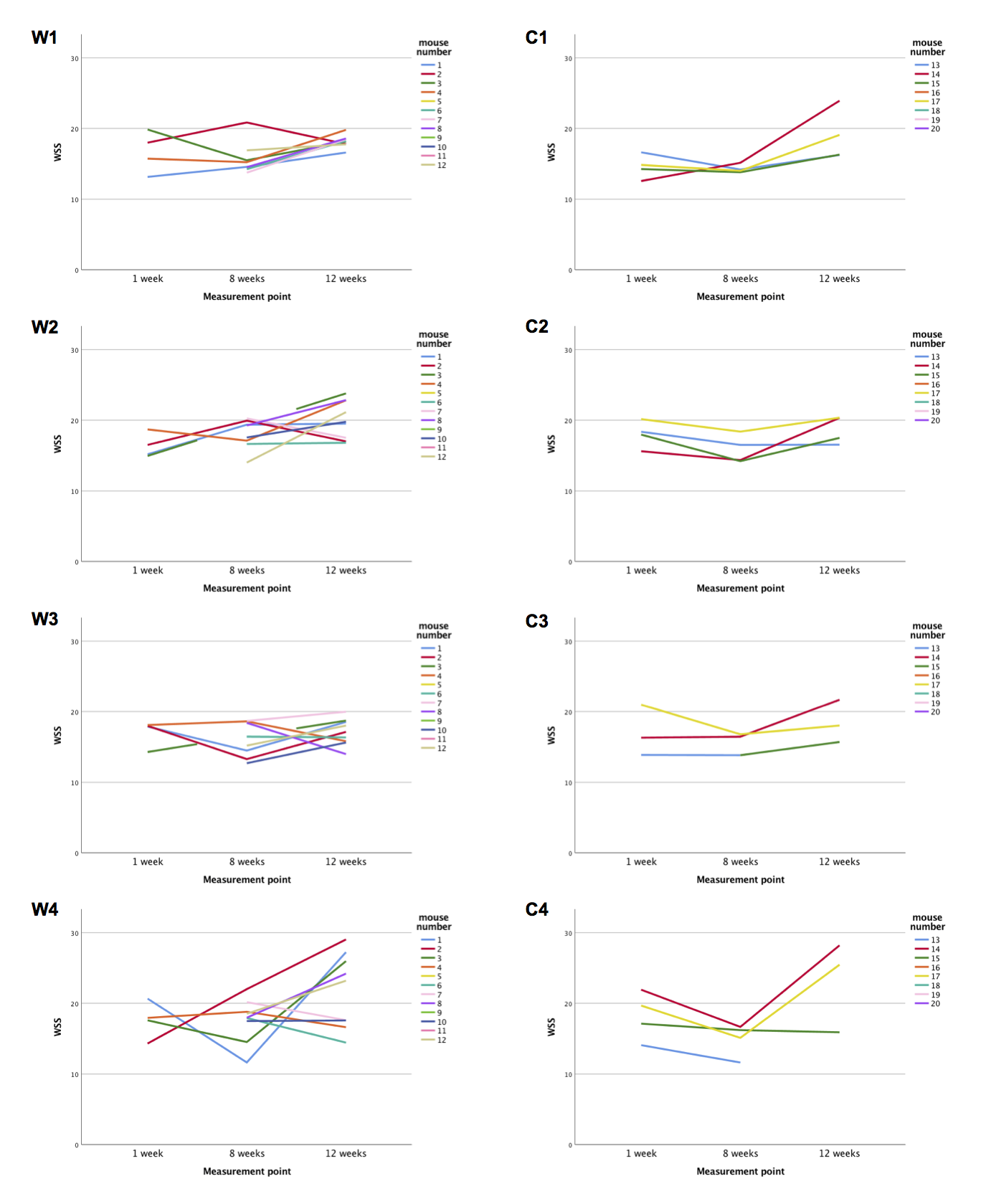

Supplement: S4 Fig — W = Western Diet, C = Chow Diet, 1–4 = MR planes 1–4. (TIFF) [file pone.0238112.s004.tiff]

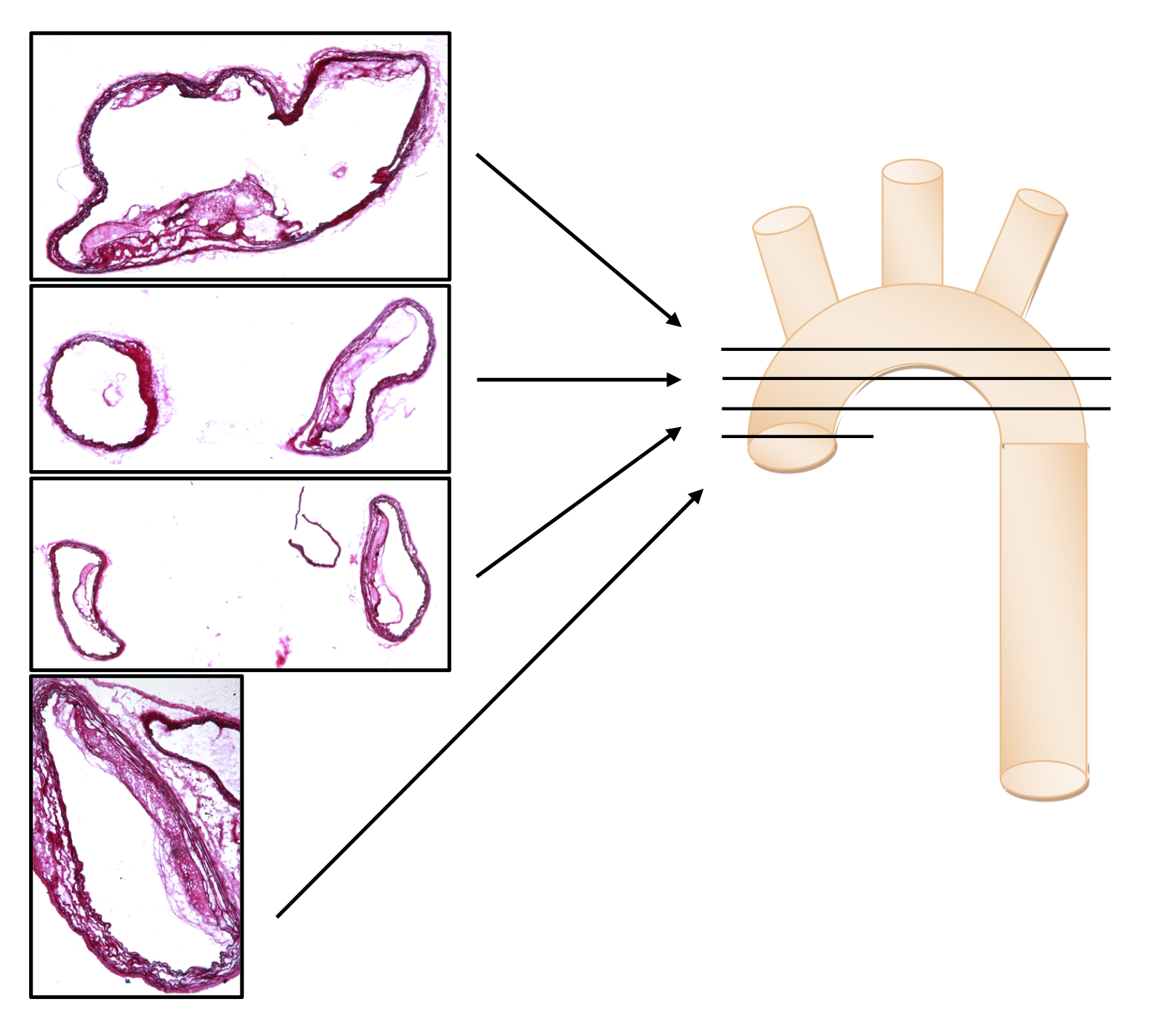

Supplement: S5 Fig — (TIFF) [file pone.0238112.s005.tiff]

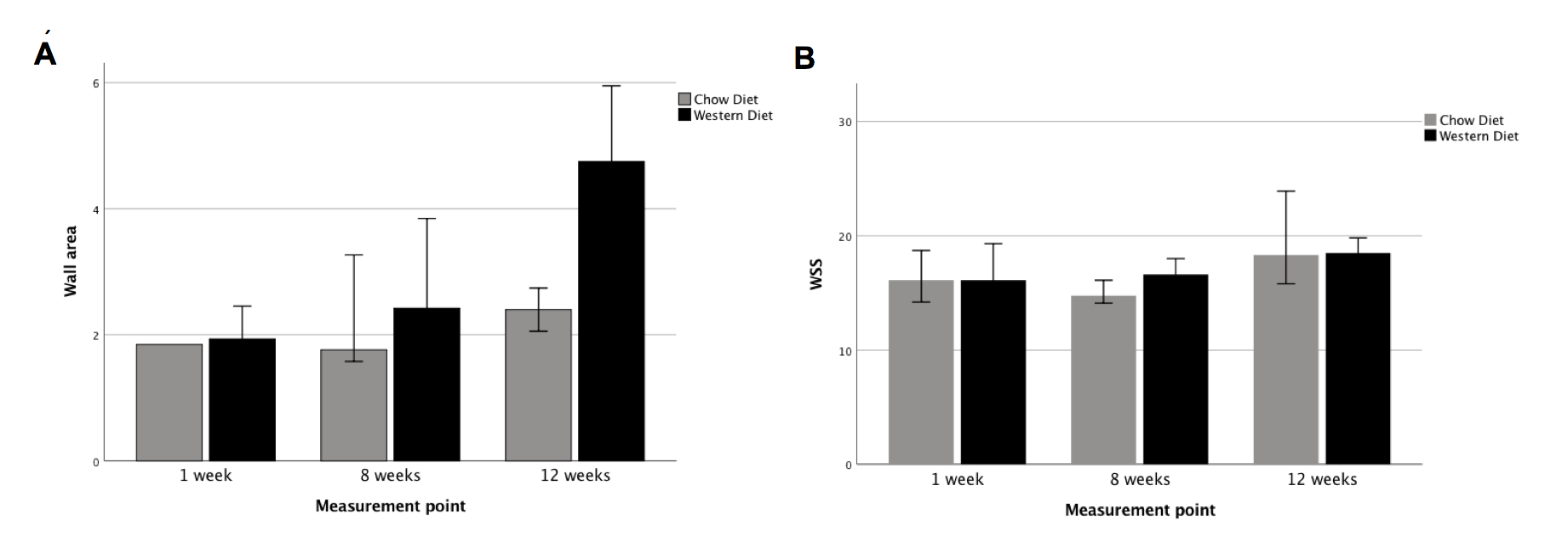

Supplement: S6 Fig — A: wall area (outer diameter minus inner diameter of the aorta) in mm2 and B: wall shear stress in N/m2. (TIFF) [file pone.0238112.s006.tiff]

**A**

| Volume V [ml] | Time t [s] | Flow V/t [ml/s] |
|---------------|------------|-----------------|
| 100           | 34.18      | 2.926           |
| 100           | 34.65      | 2.886           |
| 100           | 33.92      | 2.948           |
| 100           | 34.15      | 2.928           |
| 100           | 35.53      | 2.815           |
| 100           | 33.49      | 2.986           |
| 100           | 33.83      | 2.956           |
| 100           | 35.28      | 2.834           |
| 100           | 34.54      | 2.895           |

**B**

| MR section plane | Diameter [mm] | Flow [ml/s] |
|------------------|---------------|-------------|
| +4               | 3.870         | 2.858       |
| +3               | 3.897         | 2.996       |
| +2               | 3.875         | 3.096       |
| +1               | 3.877         | 3.081       |
| 0                | 3.861         | 3.151       |
| -1               | 3.891         | 2.882       |
| -2               | 3.894         | 2.882       |
| -3               | 3.888         | 2.946       |
| -4               | 3.868         | 2.916       |

**C**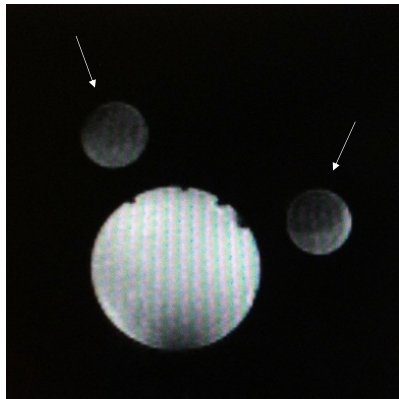

Supplement: S2 Table — A: Volume measurement, B: MR measurement and C: representative MR scan (phantom marked with white arrows, the large object is a static cylinder filled with saline water). (PDF) [file pone.0238112.s008.pdf]

**A**

| Volume V [ml] | Time t [s] | Flow V/t [ml/s] |
|---------------|------------|-----------------|
| 100           | 456.62     | 0.219           |
| 100           | 452.49     | 0.221           |
| 100           | 450.45     | 0.222           |
| 100           | 454.55     | 0.220           |
| 100           | 446.43     | 0.224           |
| 100           | 456.62     | 0.219           |
| 100           | 452.49     | 0.221           |
| 100           | 458.72     | 0.218           |
| 100           | 446.43     | 0.224           |
| 100           | 462.96     | 0.216           |

**B**

| MR section plane | Diameter [mm] | Flow [ml/s] |
|------------------|---------------|-------------|
| 1                | 0.975         | 0.206       |
| 2                | 0.972         | 0.216       |
| 3                | 0.968         | 0.220       |
| 4                | 0.978         | 0.216       |

**C**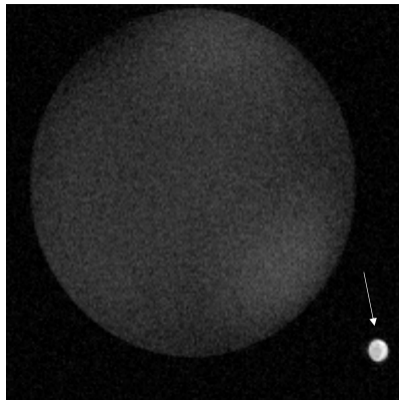

Supplement: S3 Table — A: Volume measurement, B: MR measurement and C: representative MR scan (phantom marked with white arrow, the large object is a static cylinder filled with saline water). (PDF) [file pone.0238112.s009.pdf]
