# Supplementary material for: Wall shear stress analysis using 17.6 Tesla MRI: A longitudinal study in ApoE-/- mice with histological analysis
Source: PLoS One. 2020 Aug 28;15(8):e0238112. doi: 10.1371/journal.pone.0238112 (PMC7454980; doi:10.1371/journal.pone.0238112)
Supplement: S1 Table — MR = MR measurement, Histo = histological analysis with euthanization, Exitus = death during MR measurement. (PDF) [file pone.0238112.s007.pdf]

	Mice number	1. Measurement point (1 week)	2. Measurement point (8 weeks)	3. Measurement point (12 weeks)
**Western Diet**	**1**	MR	MR	MR → Histo
	**2**	MR	MR	MR → Histo
	**3**	MR	MR	MR
	**4**	MR	MR	MR → Histo
	**5**	MR→ Histo		
	**6**		MR	MR
	**7**		MR	MR → Histo
	**8**		MR	MR
	**9**		MR → Histo	
	**10**		MR	MR
	**11**		MR	
	**12**		MR	MR
**Chow Diet**	**13**	MR	MR	MR
	**14**	MR	MR	MR
	**15**	MR	MR	MR
	**16**	MR → Histo		
	**17**	MR	MR	MR → Histo
	**18**		MR → Histo	
	**19**		MR → Exitus	
	**20**		MR → Histo	
